# Long-Term Daytime Warming Rather Than Nighttime Warming Alters Soil Microbial Composition in a Semi-Arid Grassland

**DOI:** 10.3390/biology12050699

**Published:** 2023-05-10

**Authors:** Jiayin Feng, Jingyi Ru, Jian Song, Xueli Qiu, Shiqiang Wan

**Affiliations:** 1 School of Life Sciences, Hebei University, Baoding 071002, China; 2 Institute of Life Science and Green Development, Hebei University, Baoding 071002, China

**Keywords:** climate change, asymmetric warming, microbial community, plant cover, carbon cycling, temperate steppe

## Abstract

**Simple Summary:**

Global mean temperature has increased by 1.07 °C since the Industrial Revolution, which arouses people’s widespread concern about climate warming. Nighttime temperatures increase faster and higher than daytime temperatures around the world. Various microbial groups in the soil may respond differently to such asymmetrically diurnal warming and then change ecological functions. In this study, we used a ten-year experiment in a semi-arid grassland to examine the effects of daytime and nighttime warming on soil microbial composition. The results showed that short-term warming did not change soil microbial composition, but long-term daytime warming rather than nighttime warming decreased the fungi-to-bacteria ratio in soils. In addition, soil respiration enhanced with the decreasing fungi-to-bacteria ratio. This work implies the importance of soil microbial composition in regulating grassland C release under long-term warming, which may help us accurately assess the climate-C feedback in terrestrial ecosystems.

**Abstract:**

Climate warming has profoundly influenced community structure and ecosystem functions in the terrestrial biosphere. However, how asymmetric rising temperatures between daytime and nighttime affect soil microbial communities that predominantly regulate soil carbon (C) release remains unclear. As part of a decade-long warming manipulation experiment in a semi-arid grassland, we aimed to examine the effects of short- and long-term asymmetrically diurnal warming on soil microbial composition. Neither daytime nor nighttime warming affected soil microbial composition in the short term, whereas long-term daytime warming instead of nighttime warming decreased fungal abundance by 6.28% (*p* < 0.05) and the ratio of fungi to bacteria by 6.76% (*p* < 0.01), which could be caused by the elevated soil temperature, reduced soil moisture, and increased grass cover. In addition, soil respiration enhanced with the decreasing fungi-to-bacteria ratio, but was not correlated with microbial biomass C during the 10 years, indicating that microbial composition may be more important than biomass in modulating soil respiration. These observations highlight the crucial role of soil microbial composition in regulating grassland C release under long-term climate warming, which facilitates an accurate assessment of climate-C feedback in the terrestrial biosphere.

## 1. Introduction

Climate warming has led to an increase in global mean temperature by 1.07 °C since 1850 [1] and can profoundly influence the carbon (C) balance between plants and soils in terrestrial ecosystems, resulting in an uncertain response of soil C storage to climate warming [2,3]. Soil microorganisms, an essential component in ecological communities, are extensively involved in soil organic matter decomposition and nutrient cycles, thus playing crucial roles in maintaining ecosystem stability [4,5]. Given the high sensitivity of microbial activities to rising temperatures, climate warming may greatly change the community structure and functions of soil microorganisms [6,7]. Previous studies have suggested that warming stimulates the microbe-mediated soil organic C mineralization, thus causing decreases in soil C storage [8,9]. However, some studies show conflicting outcomes and propose our superficial cognition of warming effects on soil microbial communities and C cycling [10]. Due to the fact that soil microorganisms predominantly regulate the decomposition and formation of soil organic C, and affect soil respiration by changing their metabolic activities [10,11], understanding the variation in microbial composition and their responses to climate warming can help us make accurate predictions on the climate-C feedback in the terrestrial biosphere.

Climate warming can cause variations in soil microclimates, e.g., the increase in soil temperature and reduction in soil water content, which may stimulate the rate of organic matter decomposition or inhibit microbial growth efficiency [8,9,10], subsequently resulting in different levels of soil C loss. However, long-term ecosystem responses to warming may be different from transient responses [3,11]. The stimulatory effect of warming on soil respiration weakens over time, which could be possibly attributed to the depleted C availability, the thermal adaptation of the microbial community, and the enhanced microbial interactions [2,12,13]. In addition to temperature-mediated microbial activity, changes in plant communities, which provide substantial C inputs to soils and reshape microbial composition, may affect soil C release associated with rising temperatures [3,14]. For example, long-term warming stimulates C storage in forest soils by alleviating nitrogen limitation, or changes plant community composition towards low-productivity species to offset short-lived soil C loss [2,11]. However, how the temporal responses of soil microbial communities to climate warming, and their linkages with plant cover changes and soil C loss remain largely elusive.

A greater increasing rate in daily minimum temperature than that in daily maximum temperature has been found in different regions [15,16], and such asymmetric daytime and nighttime warming can differentially affect hydro-thermal factors, plant physiology, productivity, and ecosystem C cycling [15,16,17,18,19]. Nighttime warming-induced overcompensation of plant photosynthesis and consequent C assimilation may supply more C sources for microbial growth and activity [8,20]. In addition, the enhanced nitrogen availability and organic matter decomposition rate under nighttime warming can intensify the competition for limited nutrients between plants and soil microorganisms, leading to an increase in fungal diversity, or reductions in bacterial abundance and C use efficiency [8,21]. However, our understanding of the effects of asymmetrically diurnal warming on soil microbial communities is mainly based on some short-term (less than 5 years) field experiments, and little is known about microbial feedbacks to long-term daytime and nighttime warming.

Phospholipid fatty acid (PLFA) can be used as an efficient tool to measure microbial community composition and estimate microbial responses to environmental stress [22,23]. Although it has a low taxonomic resolution compared with current molecular methods, PLFA reflects the relative dominance of biomarkers in the soil, such as the ratios of fungi to bacteria and Gram-positive to Gram-negative bacteria [23]. As part of a 10-year (2006–2015) manipulative experiment, this study was conducted to examine the responses of soil microbial composition to short- and long-term daytime and nighttime warming in a semi-arid grassland. The following two hypotheses were specifically tested: (1) long-term warming could have greater impacts on soil microbial composition than short-term warming, likely due to the changes in soil microclimate and plant cover which can reshape the soil microbial community; (2) nighttime warming would affect more microbial groups than daytime warming because the stronger response of plants to nighttime warming can further alter the soil environment and the microbial community.

## 2. Materials and Methods

### 2.1. Site Description

The study was conducted in a semi-arid grassland in Duolun County (42°02′ N, 116°17′ E, 1324 m a.s.l.), Inner Mongolia, China. Mean annual precipitation and air temperature are 374.5 mm and 2.4 °C, respectively. Sandy soil of the experimental site is classified as Haplic Calcisols according to the Food and Agriculture Organization of the United Nations (FAO) classification standard. Surface soil bulk density and pH are 1.31 g·cm^−3^ and 7.7 [24]. Plant species in this grassland are dominated by grasses (*Agropyron cristatum*, *Cleistogenes squarrosa*, *Leymus chinensis*, and *Stipa krylovii*), forbs (*Artemisia frigida*, *Potentilla tanacetifolia*, *Heteropappus altaicus*, and *Potentilla acaulis*) and legumes (*Melilotoides ruthenica*, *Astragalus adsurgens*, *and Lespedeza bicolor*).

### 2.2. Experimental Design

The experiment was established in March 2006 and conducted as part of a complete random block design with 6 treatments, including control (C), daytime warming (6:00–18:00; D), nighttime warming (18:00–6:00; N), diurnal warming (24 h; W), nitrogen fertilization, and diurnal warming plus nitrogen fertilization. There were 4 replicates for each treatment, and therefore 24 3 m × 4 m plots were assigned in a 4 × 6 matrix, with a 3 m distance between adjacent plots.

The infrared radiators (165 cm × 15 cm; MSR-2420, Kalglo Electronics, Bethlehem, PA, USA) were placed 2.25 m above the ground and set at 1600W as the power output in each warming plot [19,25]. The daily mean soil temperatures were elevated by 0.91 °C and 1.18 °C under daytime and nighttime warming, respectively (Figure S1). In each control plot, a “dummy’’ heater with the same shape and size as the infrared heater was installed at the same height to imitate the shading effect. The asymmetric diurnal warming treatments began in April 2006, and the heaters ran continuously and automatically from 15 March to 15 November in each year. No other manipulations were performed in the experimental plots.

### 2.3. Soil Microclimate, PLFA Analysis, and Plant Cover Measurement

Soil temperature at the depth of 10 cm was automatically recorded every 10 min using a STM-01 Soil Temperature Measurement System Datalogger (Henan Electronic Institute, China), and soil temperatures during the growing season from May to October were used as the annual mean value [19]. Soil moisture (SM) at the same site and depth was measured by a portable soil moisture probe (Diviner-2000, Sentek, Australia). Soil moisture was analyzed three times per month from May to October each year. 

A total of 2 soil cores (0–10 cm) were randomly collected using a soil auger in late August in 2006, 2007, 2011, 2012, and 2015. Soil samples in each plot were mixed together after removing visible extraneous materials and passing through a 2 mm sieve, and then stored at −20 °C until phospholipid fatty acid (PLFA) extraction. In brief, 8 g of dry soil were fractionated and quantified with a mixture containing chloroform, methanol, and phosphate buffer (1:2:0.8 *v*/*v*/*v*). The lipids were filtrated with silicic acid column and separated into phospholipids, glycolipids, and neutral lipids, and then subjected to a mild alkaline methanolysis. Then, 19:0 methyl esters were dissolved in hexane as an internal standard. Extracted fatty acid methyl esters were identified with gas chromatograph (Agilent 7890A, Agilent technologies, Santa Clara, CA, USA) and a microbial identification system (MIDI Inc., Newark, DE, USA). The relative biomass of fungi was determined by the summed concentrations of 18:1ω9c, 18:2ω6c, and 16:1ω5c. Gram-negative (G−) bacteria biomass was estimated by 16:1 2OH, 16:1ω7, 16:1ω9, 17:0 cyclo, 17:1ω8, 18:1ω5, 18:1ω7, and 19:0 cyclo ω8, while Gram-positive (G+) bacteria contained 15:0 anteiso, 15:0 iso, 16:0 iso, 16:1 iso G, 17:0 anteiso, 17:0 iso, 18:1ω7 11-methyl, 18:1ω9, and 19:1ω11. The summed concentrations of general FAME, G−, and G+ bacteria were identified as bacterial biomarkers [5]. Specific methodologies for determining microbial biomass C and soil respiration can be found in the Supplementary Materials. The percent cover of each plant species in each 1 m × 1 m plot was recorded by visual estimation in late August each year.

### 2.4. Data Analysis

The effects of asymmetric daytime and nighttime warming and year, and their possible interactions on soil microclimate and microbial compositions were analyzed using two-way repeated ANOVA measures with SAS V8 software (SAS Institute). Linear regressions were investigated to determine correlations among soil microbial compositions, soil microclimate, and plant functional groups. Redundancy analysis (RDA) and random forest modeling were performed to reveal environmental factors influencing microbial composition in R (v 3.4.0). Structural equation modeling (SEM) exploring the effects of abiotic and biotic factors on soil microbial composition was constructed using AMOS 24.0 (Amos Development Corporation). The goodness of model fit was tested based on the *p*-value (>0.05), chi-square, and root mean square error of approximation (RMSEA; <0.05). The total, direct, and indirect effects of the abiotic and biotic factors on soil microbial composition were also recorded.

## 3. Results

### 3.1. Soil Microclimate

**Figure 1:**
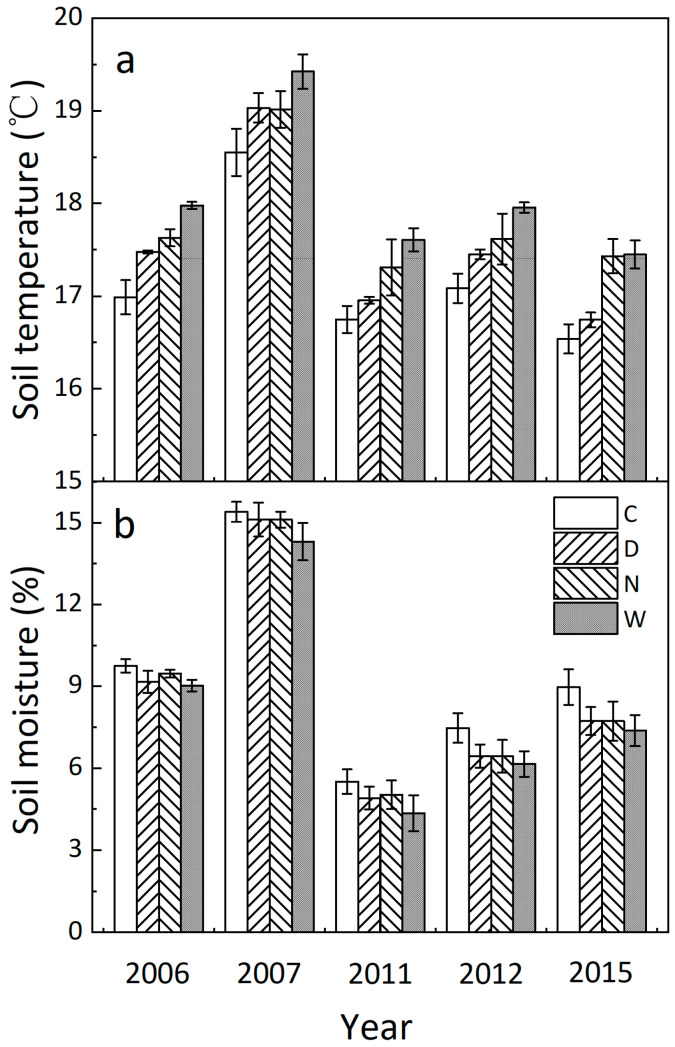
Annual mean soil temperature (**a**) and moisture (**b**) under the 4 treatments in 2006, 2007, 2011, 2012, and 2015 (M ± 1 SE, n = 4). C, control; D, daytime warming; N, nighttime warming; and W, diurnal warming.

Averaged across the 4 treatments, both soil temperature and moisture substantially fluctuated over the experimental durations (Figure 1 and Figure S2, both *p* < 0.001, Table 1). Daily mean soil temperature was enhanced by 0.32 °C and 0.58 °C under daytime and nighttime warming averaged over the 5 years, respectively (Figure 1a, both *p* < 0.001). Soil moisture was decreased by 0.65% (absolute change, *p* < 0.01) and 0.55% (*p* < 0.05) under daytime and nighttime warming, respectively (Figure 1b). There was no interaction between daytime and nighttime warming on soil temperature or moisture.

When separating into the short- (2006 and 2007) and long-term (2011, 2012, and 2015) stages, daytime warming significantly increased soil temperature by 0.43° (*p* < 0.001) and marginally reduced soil moisture by 0.53% (*p* < 0.1). Nighttime warming stimulated soil temperature by 0.50 °C (*p* < 0.001) but had no effect on soil moisture (*p* > 0.05) in the short term (Figure 1 and Table 1). By contrast, daytime warming enhanced soil temperature by 0.23 °C (*p* < 0.05) and suppressed soil moisture by 0.70% (*p* < 0.05), and nighttime warming increased soil temperature by 0.64 °C (*p* < 0.001) and decreased soil moisture by 0.66% (*p* < 0.05) in the long term (Figure 1).

### 3.2. Soil Microbial Composition

**Figure 2:**
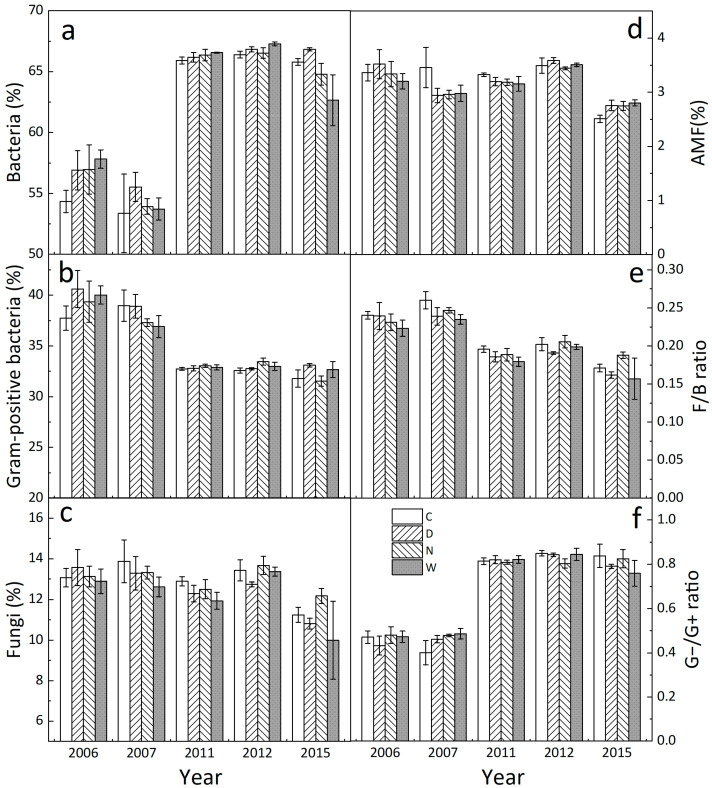
Annual mean relative abundance of bacteria (**a**), Gram-positive bacteria (**b**), fungi (**c**), arbuscular mycorrhizal fungi (AMF, (**d**)), fungi-to-bacteria ratio (F/B ratio) (**e**), and Gram-negative bacteria to Gram-positive bacteria ratio (G−/G+ ratio) (**f**) under the 4 treatments in 2006, 2007, 2011, 2012, and 2015 (M ± 1 SE, n = 4). See Figure 1 for abbreviations.

Averaged over the 5 years, daytime warming did not affect the relative abundance of bacteria, G+ bacteria, AMF, or the ratio of G− to G+ bacteria (Figure 2a,b,d,f, all *p* > 0.05, Table 1), but reduced fungi proportion by 4.47% (Figure 2c, *p* < 0.05) and the fungi-to-bacteria ratio by 5.63% (Figure 2e, *p* < 0.01). Nighttime warming had no effect on the relative abundance of any of the microbial groups or the ratios of fungi to bacteria and G− to G+ bacteria (Figure 2, all *p* > 0.05). The responses of microbial compositions to daytime and nighttime warming varied with year (all *p* < 0.001). No interaction of daytime and nighttime warming on the relative abundance of any of microbial groups or the ratios of fungi to bacteria and G− to G+ bacteria was detected. 

**Figure 3:**
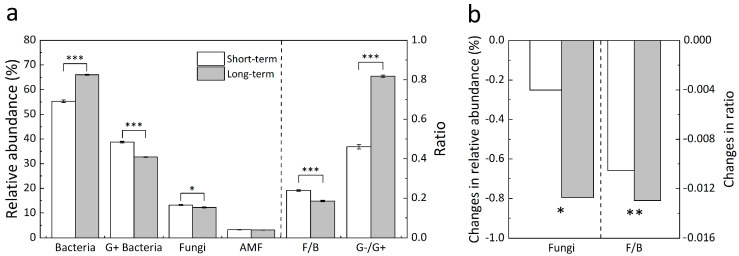
Comparison of microbial compositions between short- (2006 and 2007) and long-term (2011, 2012, and 2015) warming (**a**) and daytime warming-induced changes in the relative abundance of fungi and the fungi-to-bacteria ratio (F/B) (**b**). Each blank column in Panel a represents the mean value of 2006 and 2007, and each gray column in *Panel a* represents the mean value of 2011, 2012, and 2015. Significance levels: * *p* < 0.05, ** *p* < 0.01, and *** *p* < 0.001.

Averaged across the four treatments, the relative abundance of bacteria and the G− to G+ bacteria ratio were higher in the long- than in the short-term stage (Figure 3a, all *p* < 0.001). However, the relative abundance of G+ bacteria (*p* < 0.001) and fungi (*p* < 0.05) as well as the fungi-to-bacteria ratio (*p* < 0.001) in the long term were lower than those in the short term (Figure 3a). Nighttime warming did not change the composition of any of the microbial groups in either short or long terms (all *p* > 0.05, Table 1), whereas daytime warming reduced the relative abundance of fungi by 6.28% (*p* < 0.05) and the ratio of fungi to bacteria by 6.76% (*p* < 0.01) in the long term only (Figure 3b).

### 3.3. Relationships of Microbial Composition with Soil Microclimate and Plant Cover 

Grass cover was not affected by daytime warming but was significantly reduced by 19.2% (Figure S3a, *p* < 0.05, Table S1) under nighttime warming in the long term. Forb cover was suppressed by 8.89% and 22.5% (both *p* < 0.05) under daytime warming in the short and long term, respectively, but remained unchanged under nighttime warming (Figure S3b). Legume cover was increased by 82.8% (*p* < 0.01) under daytime warming in the short term but was not changed under nighttime warming (Figure S3c).

**Figure 4:**
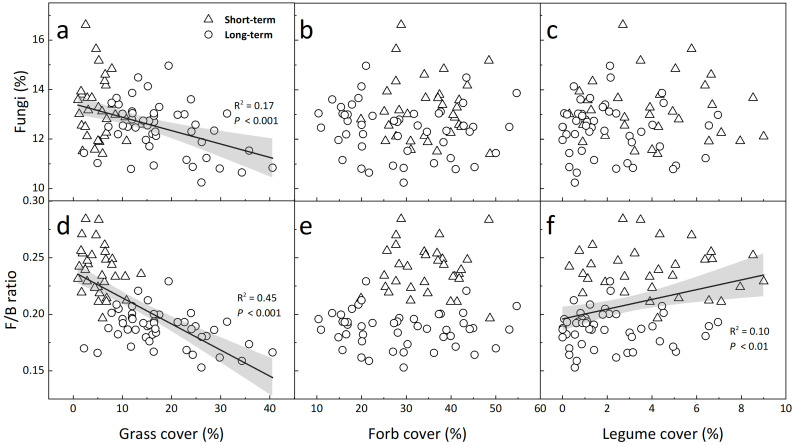
Dependences of the relative abundance of fungi (**a**–**c**) and the ratios of fungi to bacteria (**d**–**f**) on plant cover of grass (**a**,**d**), forb (**b**,**e**), and legume (**c**,**f**). Each data point represents the mean annual value in each plot in the short (2006 and 2007) and long term (2011, 2012, and 2015). The solid lines and shaded areas describe linear regressions and 95% CIs.

The relative abundance of G+ bacteria positively depended on soil moisture (Figure S4a, R^2^ = 0.32, *p* < 0.001), but negatively related to grass cover (Figure S4b, R^2^ = 0.34, *p* < 0.001), whereas G− bacteria linearly reduced with increasing soil moisture (Figure S4e, R^2^ = 0.60, *p* < 0.001) and legume cover (Figure S4h, R^2^ = 0.21, *p* < 0.001), but enhanced with increasing grass cover (Figure S4f, R^2^ = 0.39, *p* < 0.001). The relative abundance of fungi showed negative linear dependence on grass cover (Figure 4a, R^2^ = 0.16, *p* < 0.001) over the 5 years, but was not related to forb or legume cover (Figure 4b,c, both *p* > 0.05). The ratio of fungi to bacteria linearly decreased with grass cover (Figure 4d, R^2^ = 0.45, *p* < 0.001) and increased with legume cover (Figure 4f, R^2^ = 0.09, *p* < 0.01), but was not correlated with forb cover (Figure 4e, *p* > 0.05). Both the relative abundance of fungi (Figure S6a, R^2^ = 0.12, *p* < 0.001) and fungi-to-bacteria ratio (Figure S6c, R^2^ = 0.37, *p* < 0.001) positively depended upon soil temperature, but only the fungi-to-bacteria ratio was positively related to soil moisture (Figure S6d, R^2^ = 0.39, *p* < 0.001).

### 3.4. Controls of Abiotic and Biotic Factors on Soil Microbial Composition

**Figure 5:**
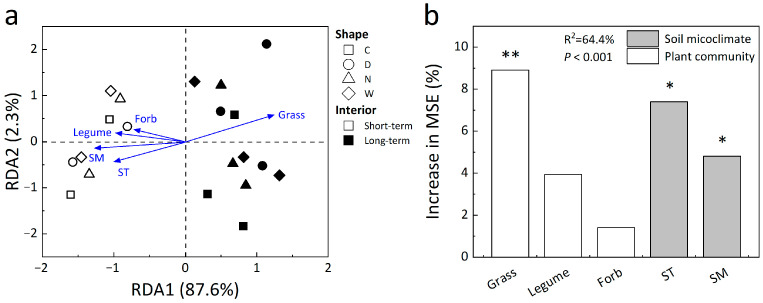
Redundancy analysis (RDA) (**a**) among soil temperature (ST), soil moisture (SM), plant (grass, forb, and legume) cover and different treatments, and random forest analysis (**b**) for important predictors of the fungi-to-bacteria ratio. ST, soil temperature; SM, soil moisture. Significance levels: * *p* < 0.05, ** *p* < 0.01. See Figure 1 for abbreviations.

Redundancy analysis (RDA) revealed the correlations among different treatments, soil microclimate, and plant functional groups, with the first and second canonical axis explaining 87.6% and 2.3% of the variance, respectively (Figure 5a). Grass cover, soil temperature, and moisture were the dominant factors influencing microbial variations, accounting for 8.91% (*p* < 0.01), 7.40% (*p* < 0.05), and 4.81% (*p* < 0.05) of the increase in mean square error (InMSE) based on random forest model analysis (Figure 5b).

**Figure 6:**
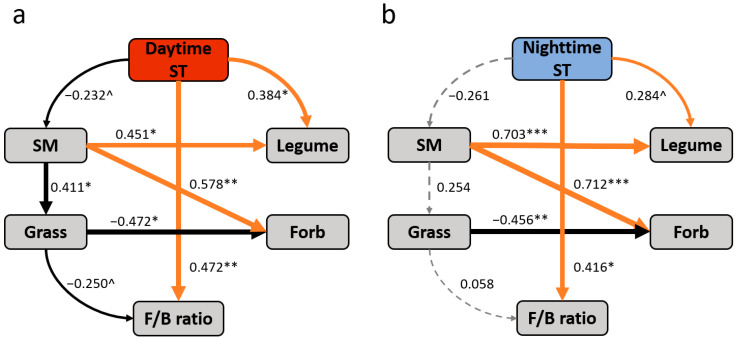
Structural equation models of the direct and indirect effects of daytime soil temperature on the fungi-to-bacteria ratio under daytime warming (**a**) and nighttime soil temperature on the fungi-to-bacteria ratio under nighttime warming (**b**). The solid and dashed arrows represent significant (*p* < 0.1) and non-significant (*p* > 0.1) paths. Orange and black arrows represent positive and negative paths. The width of the arrow indicates the strength of the relationship. Numbers adjacent to arrows are standardized path coefficients and are indicative of the effect size of the relationships. The final model fits the data well, as suggested by the chi-square and RMSEA values ((**a**), χ^2^ = 4.622, *p* = 0.706, RMSEA = 0, *df* = 7; (**b**), χ^2^ = 4.035, *p* = 0.776, RMSEA = 0, *df* = 7). Significance levels: ^ *p* < 0.1, * *p* < 0.05, ** *p* < 0.01, *** *p* < 0.001. See Figure 1 and Figure 5 for abbreviations.

The results of SEM analysis showed that under daytime warming, the increased soil temperature directly changed microbial composition as revealed by the fungi-to-bacteria ratio (standardized direct effect was 0.472, Figure 6a), and indirectly affected it through the changes in soil moisture and grass cover (standardized indirect effect was 0.024, Figure 6a). Under nighttime warming, the ratio of fungi to bacteria was significantly correlated with nighttime soil temperature only (standardized total effect was 0.412, Figure 6b).

## 4. Discussion

### 4.1. Short-and Long-Term Warming on Soil Microbial Composition

The obvious interannual variation in soil microclimate and some microbial groups (e.g., G+ and G− bacteria) found in our study can be mainly attributed to the changes in precipitation amounts, the natural succession of plant communities, and the potential interactions among different microbial components (Figures S2–S5). Our results that long-term rather than short-term warming decreases fungal abundance and alters microbial composition revealed by the fungi-to-bacteria ratio agree with previous findings that warming effects on soil microbial communities mainly depend on treatment duration [9,26], with unchanged microbial biomass, diversity, and composition observing in several short-term (e.g., 1–6 years) warming experiments [9,26,27]. This can be explained by several possible reasons: First, the moderate elevation of soil temperatures (<1 °C) during the first two years found in our study might not be sufficient to induce a significant response in the microbial community due to the wide range of microbial adaptation [9,26]. Second, although soil moisture, forb, and legume cover vary with short-term warming, they may have legacy effects on the soil microbial community because of the strong resistance and functional redundancy [8,9]. Third, the assembly and stabilization of the soil microbial community experienced considerable long-term environmental fluctuations, which partially explains the weak response of soil microorganisms to short-term warming [28]. 

In addition, the reductions in fungal abundance and consequent fungi-to-bacteria ratio under long-term warming can be attributed to the changes in soil microclimate and increased cover of grasses (Figure 4, Figure 5, Figures S6 and S8), which agrees with our Hypothesis 1. Previous studies have found the suppressed competitive ability of some grasses decreased microbial biomass and C use efficiency under warming in this temperate steppe [8,19], but no linkages between above- and below-ground components were examined. The dominant grass species in this grassland, such as *Achnatherum Sibiricum*, *Agropyron Cristatum*, and *Stipa Krylovii*, typically have taller stature but shallower root distributions than forbs and legumes; therefore, the presence of these species may lead to lower soil temperature because of the shading effect and have subsequent impacts on microbial activity in surface soils. Moreover, warming can reduce organic C inputs through grass litter [29] which decreases substrate availability, thereby decreasing fungal activity and saprotrophic fungal proportions [7]. Overall, our study not only suggests the close association between soil microbial composition and grass cover but also indicates that fungi respond to long-term warming more sensitively than bacteria in this semi-arid grassland.

### 4.2. Asymmetrically Diurnal Warming on Soil Microbial Composition

The observations of decreased fungal abundance and the fungi-to-bacteria ratio under daytime warming in the long term do not agree with our Hypothesis 2 and previous findings that nighttime warming increases fungal diversity and changes microbial composition and C use efficiency [8,19,21]. Two potential mechanisms may account for the daytime warming-induced reduction in fungal abundance. First, the enhancement in microbial metabolism and water consumption under daytime warming might lead to rapid exhaustion of C substrates, thus inducing an inhibition of microbial activity. Given that soil fungi, especially the saprotrophic lineages, are important decomposers of soil organic matter [30], the decreased water can significantly suppress the abundance of the fungal community. Second, because of the key role of plant growth in triggering microbial variations, the competition between root absorption and microbial activity for nutrients in soils may limit the growth of fungi which usually colonize plant rhizospheres [8,31]. Nighttime warming-induced overcompensation of plant photosynthesis also improves the substrates’ utilization of plant roots in the daytime and further reduces fungal abundance [8,15,19,20].

The stronger impacts of warming on fungi than bacteria have been widely reported [32,33]. One possibility is that most fungi have higher C assimilation efficiency and metabolic ability than bacteria, hence the warming-induced decrease in soil water availability could significantly inhibit the growth and activity of fungi, showing greater suppression on the biomass and diversity of fungi, especially in our experimental grassland which is largely water limited [23]. A reduction in plant litter residues with higher cellulose contents under warming can also lead to a decline in saprotrophic fungal abundance [34,35]. In addition, although AMF is important in facilitating plant nutrient absorption and usually shows sensitive responses to warming [36], the unchanged AMF abundance observed in our study might contribute to their different temperature optima [37] and the direct nutrient acquisition process because they are obligate symbionts that can receive growth substrates from host plants.

### 4.3. Implications

**Figure 7:**
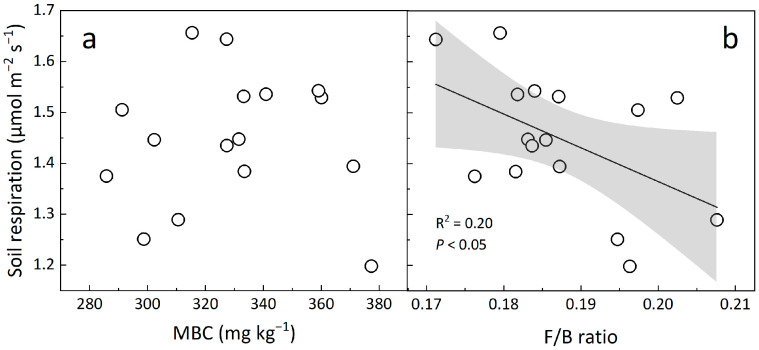
Dependence of soil respiration on microbial biomass C (MBC, (**a**)) and the fungi-to-bacteria ratio (F/B ratio, (**b**)) across spatial scales. Each data point represents the 5-year mean value of each plot. The solid lines and shaded areas describe linear regression and 95% CIs.

Climate warming can result in soil C loss (release more CO_2_ to the atmosphere) by stimulating microbial respiration and improving microbe-mediated mineralization of soil C stocks [35,38]. As the important component of soil respiration, microbial respiration is always thought to be closely associated with their biomass in most studies. However, the insignificant correlation between soil respiration and microbial biomass C, and the negative dependence of soil respiration on the fungi-to-bacteria ratio observed in our study suggest that microbial composition may be more important than biomass in regulating soil respiration under long-term warming (Figure 7). These findings indicate that changes in microbial composition in response to climate warming are critical for ecosystem C cycling in the semi-arid grassland and can provide new insights to predict the climate-C feedback in terrestrial ecosystems.

## 5. Conclusions

Climate warming has both direct and indirect effects on soil microbial composition with implications for soil respiration in the temperate steppe. Our study shows that long-term rather than short-term warming has significant impacts on soil microbial composition, which can be attributed to the changes in soil microclimate and grass cover. The decreases in fungal abundance and the fungi-to-bacteria ratio under long-term daytime warming instead of nighttime warming observed in this study are also mainly driven by the increased soil temperature, reduced soil moisture, and improved grass cover. Our findings indicate that microbial composition may be more important than microbial biomass in regulating soil respiration under long-term climate warming, which will facilitate an accurate assessment of climate warming-C feedback in terrestrial ecosystems.

## Figures and Tables

**Table 1:** Results (*F*-values) of repeated measures ANOVA on the effects of daytime warming (D), nighttime warming (N), and year (Y) and their interactions on soil temperature (ST), soil moisture (SM), the relative abundance of bacteria, Gram-negative (G+) bacteria, fungi, arbuscular mycorrhizal fungi (AMF), and the ratios of fungi to bacteria (F/B ratio) and G− to G+ bacteria (G−/G+ ratio) in all 5 years, short (2006 and 2007) and long (2011, 2012 and 2015) term.

Source of Variations		ST	SM	Bacteria	G+ Bacteria	Fungi	AMF	F/B Ratio	G−/G+ Ratio
All 5 years	D	18.6 ***	7.85 **	1.28	1.54	3.76 *	0.06	7.53 **	0.08
	N	64.1 ***	5.94 *	0.08	0.20	0.29	1.57	0.53	0.15
	Y	93.3 ***	233 ***	104 ***	53.1 ***	8.30 ***	17.3 ***	38.8 ***	166 ***
	D × N	0.20	0.29	1.82	0.76	0.53	0.04	0.03	0.00
	D × Y	0.70	0.05	0.52	1.01	0.60	1.01	0.47	1.38
	N × Y	0.71	0.19	1.91	1.16	0.38	0.95	0.74	1.10
	D × N × Y	0.11	0.34	0.41	0.22	0.39	1.21	0.22	0.45
Short term	D	14.3 ***	3.27 ^	1.37	0.64	0.28	0.60	2.52	0.06
	N	19.2 ***	1.67	0.25	0.48	0.93	1.84	2.02	2.55
	Y	170 ***	372 ***	4.26 *	2.17	0.05	3.37 ^	2.02	0.09
	D × N	0.20	0.10	0.80	0.43	0.19	0.17	0.01	0.12
	D × Y	0.01	0.00	0.10	1.08	0.68	0.77	0.57	1.48
	N × Y	0.39	0.33	1.08	1.48	0.10	0.04	0.17	0.39
	D × N × Y	0.02	0.32	0.02	0.24	0.11	1.96	0.06	0.80
Long term	D	6.24 *	4.75 *	0.05	1.93	4.39 *	1.12	5.11 *	0.36
	N	45.5 ***	4.27 *	2.36	0.31	0.01	0.00	0.09	0.90
	Y	9.57 ***	29.5 ***	6.35 **	3.04 ^	11.8 ***	100 ***	9.65 ***	1.14
	D × N	0.05	0.68	1.42	0.51	0.35	0.18	0.09	0.12
	D × Y	0.54	0.02	0.67	3.35*	0.47	2.13	0.66	1.94
	N × Y	0.77	0.06	5.72 **	1.15	0.37	2.48	0.52	0.20
	D × N × Y	0.19	0.22	1.80	0.09	0.75	0.79	0.41	0.36

Significance levels: ^ *p* < 0.1, * *p* < 0.05, ** *p* < 0.01, and *** *p* < 0.001.

## Data Availability

The data presented in this study are available upon request from the corresponding author.

## Supplementary Materials

The following supporting information can be downloaded at: https://www.mdpi.com/article/10.3390/biology12050699/s1, Figure S1: Warming-induced changes in soil temperature from 0:00–23:00 on each day (a) and from 1 May to 31 October during the growing season in each year (b) at a depth of 10 cm. Mean soil temperature (M ± 1 SE, n = 4) during short term, long term, and all 5 years (c). C, control; D, daytime warming; N, nighttime warming; and W, diurnal warming; Figure S2: Dependences of soil moisture on precipitation amount in August in 2006, 2007, 2011, 2012, and 2015. Each data point represents the mean annual value in each plot. The solid lines and shaded areas describe linear regressions and 95% CIs; Figure S3: Annual mean grass (a), forb (b), and legume cover (c) under the 4 treatments in 2006 2007, 2011, 2012, and 2015 (M ± 1 SE, n = 4). See Figure S1 for abbreviations; Figure S4: Dependences of the relative abundance of Gram-positive (G+; a–d) and Gram-negative (G−; e–h) bacteria on soil moisture (a,e) and plant cover of grasses (b,f), forbs (c,g), and legumes (d,h). Each data point represents the mean annual value in each plot. The solid lines and shaded areas describe linear regressions and 95% CIs; Figure S5: Dependences of the relative abundance of fungi on bacteria (a), G+ bacteria (b), G− bacteria (c), and G−/G+ ratio (d). Each data point represents the mean annual value in each plot. The solid lines and shaded areas describe linear regressions and 95% CIs; Figure S6: Dependences of the relative abundance of fungi (a,b) and the ratio of fungi to bacteria (c,d) on soil temperature (a,c) and moisture (b,d). Each data point represents the mean annual value in each plot in the short (2006 and 2007) and long term (2011, 2012, and 2015). The solid lines and shaded areas describe linear regressions and 95% CIs; Figure S7: Dependences of grass (a,b), forb (c,d), and legume cover (e,f) on soil temperature (a,c,e) and moisture (b,d,f). Each data point represents the mean annual value in each plot in the short (2006 and 2007) and long term (2011, 2012, and 2015). The solid lines and shaded areas describe linear regressions and 95% CIs; Figure S8: Dependences of warming-induced changes in the relative abundance of fungi (a–e) and the ratio of fungi to bacteria (f–j) on warming-induced changes in grass cover (a,f), forb cover (b,g), legume cover (c,h), soil temperature (d,i), and moisture (e,j). Each data point represents the mean annual value in each plot in the short (2006 and 2007) and long term (2011, 2012, and 2015). The solid lines and shaded areas describe linear regressions and 95% CIs; Table S1: Results (*F*-values) of repeated measures ANOVA on the effects of daytime warming (D), nighttime warming (N), and year (Y) on plant functional community (grass, forb, and legume) cover in the short (2006 and 2007) and long (2011, 2012, and 2015) term.
