# Supplementary material for: Long-Term Daytime Warming Rather Than Nighttime Warming Alters Soil Microbial Composition in a Semi-Arid Grassland
Source: Biology (Basel). 2023 May 10;12(5):699. doi: 10.3390/biology12050699 (PMC10215784; doi:10.3390/biology12050699)

Long-Term Daytime Warming Rather than Nighttime Warming Alters Soil Microbial Composition in a Semi-Arid Grassland

Jiayin Feng [1,2], Jingyi Ru [1,2], Jian Song [1,2], Xueli Qiu [1,2] and Shiqiang Wan [1,2,*]

[1] School of Life Sciences, Hebei University, Baoding 071002, China; jyfeng@hbu.edu.cn (J.F.); rujingyi@hbu.edu.cn (J.R.); jsong@hbu.edu.cn (J.S.); qxuelihenu@163.com (X.Q.)
[2] Institute of Life Science and Green Development, Hebei University, Baoding 071002, China
* Correspondence: swan@hbu.edu.cn

S1. Measurement of soil microbial biomass C and respiration

Soil microbial biomass C (MBC) was detected using the chloroform fumigation extraction method based on previous study (Liu et al., 2016). Briefly, fresh soil samples (15 g dry weight equivalent) were fumigated for 24 h with $CHCl_3$. Both fumigated and unfumigated samples were extracted with 60 ml 0.5 M K_2SO_4 for 30 min, and filtered through 0.45-μm filters. Soil MBC was detected using an elemental analyzer (TOC, Analysensysteme, Germany) and calculated from the difference between extractable C contents in the fumigated versus the unfumigated samples using a conversion factor of 0.45.

In 2004, two PVC collars (11 cm in diameter) were permanently installed in the soil of each subplot for the soil respiration measurement. Living plants inside the collars were frequently clipped and removed from the collars. Soil respiration was measured three times a month from using a Li-8100 Portable Soil CO_2 Flux System (Li-Cor Inc.). Measurements were done between 8:30 a.m. and 11:30 a.m. (local time) and the mean values of the two collars were calculated as the soil respiration for each subplot plot.

Table S1 Results (*F*-values) of repeated measures ANOVA on the effects of daytime warming (D), nighttime warming (N) and year (Y) on plant functional community (grass, forb and legume) cover in the short (2006 and 2007) and long (2011, 2012 and 2015) term.

Source of variations		Grass	Forb	Legume
	D	1.31	4.32*	10.4**
	N	0.01	2.50	0.88
	Y	44.3***	8.90**	0.09
Short-term	D×N	1.18	0.31	5.62*
	D×Y	1.16	2.38	0.45
	N×Y	1.83	3.63^	0.06
	D×N×Y	0.68	3.24^	0.09
	D	0.76	4.94*	0.47
	N	4.17*	0.18	2.03
	Y	14.8***	2.80^	1.15
Long-term	D×N	0.57	2.20	3.09^
	D×Y	0.39	0.26	0.21
	N×Y	3.69*	1.09	1.26
	D×N×Y	0.80	0.47	0.19

Significance levels: ^$P < 0.1$, *$P < 0.05$, **$P < 0.01$, ***$P < 0.001$.

Figure S1 Warming-induced changes in soil temperature from 0:00-23:00 in each day (a) and from May 1 to October 31 during the growth season in each year (b) at the depth of 10 cm. Mean soil temperature (M ± 1 SE, n = 4) during short-term, long-term, and all 5 years (c). C, control; D, daytime warming; N, nighttime warming; W, diurnal warming.

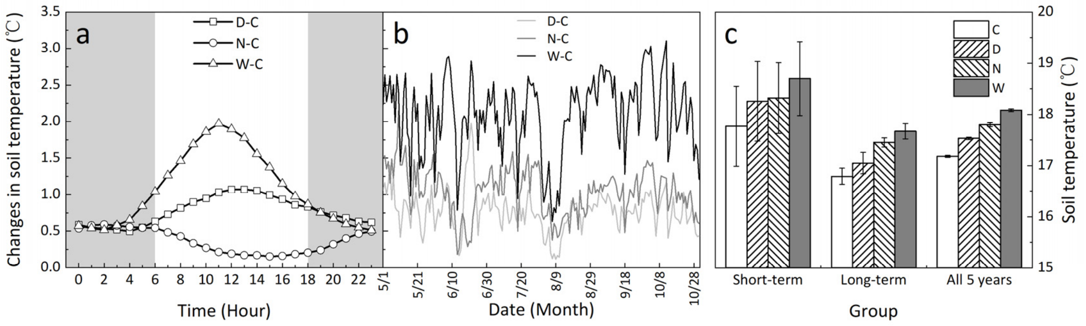

Figure S2 Dependences of soil moisture on precipitation amount in August in 2006, 2007, 2011, 2012, and 2015. Each data point represents the mean annual value in each plot. Solid line and shaded area describe linear regressions and 95% CIs.

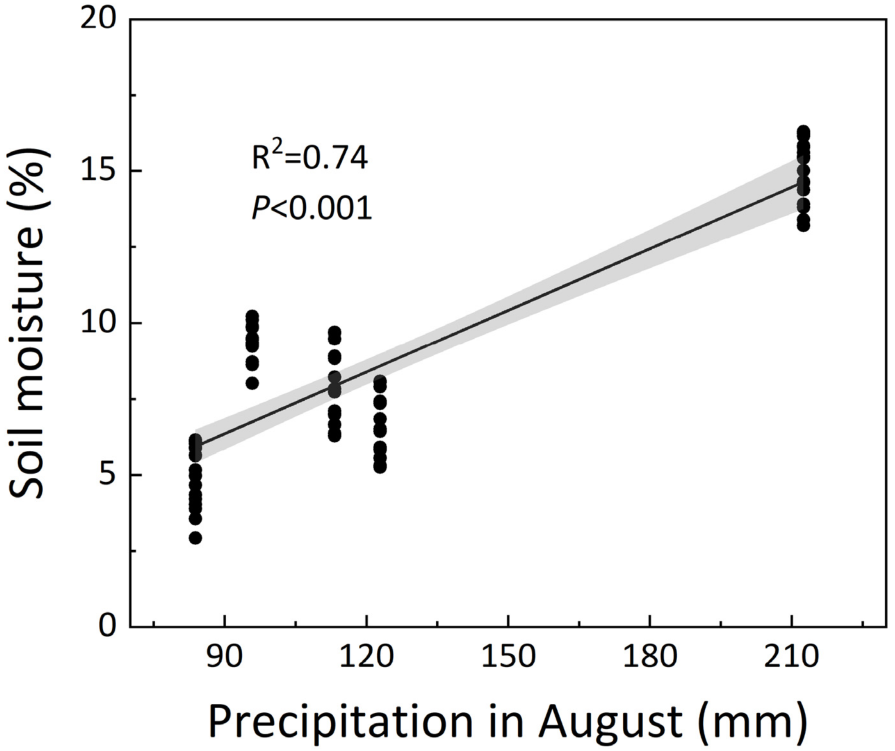

Figure S3 Annual mean grass (a), forb (b) and legume cover (c) under the four treatments in 2006 2007, 2011, 2012, and 2015 (M ± 1 SE, n = 4). See Fig. S1 for abbreviations.

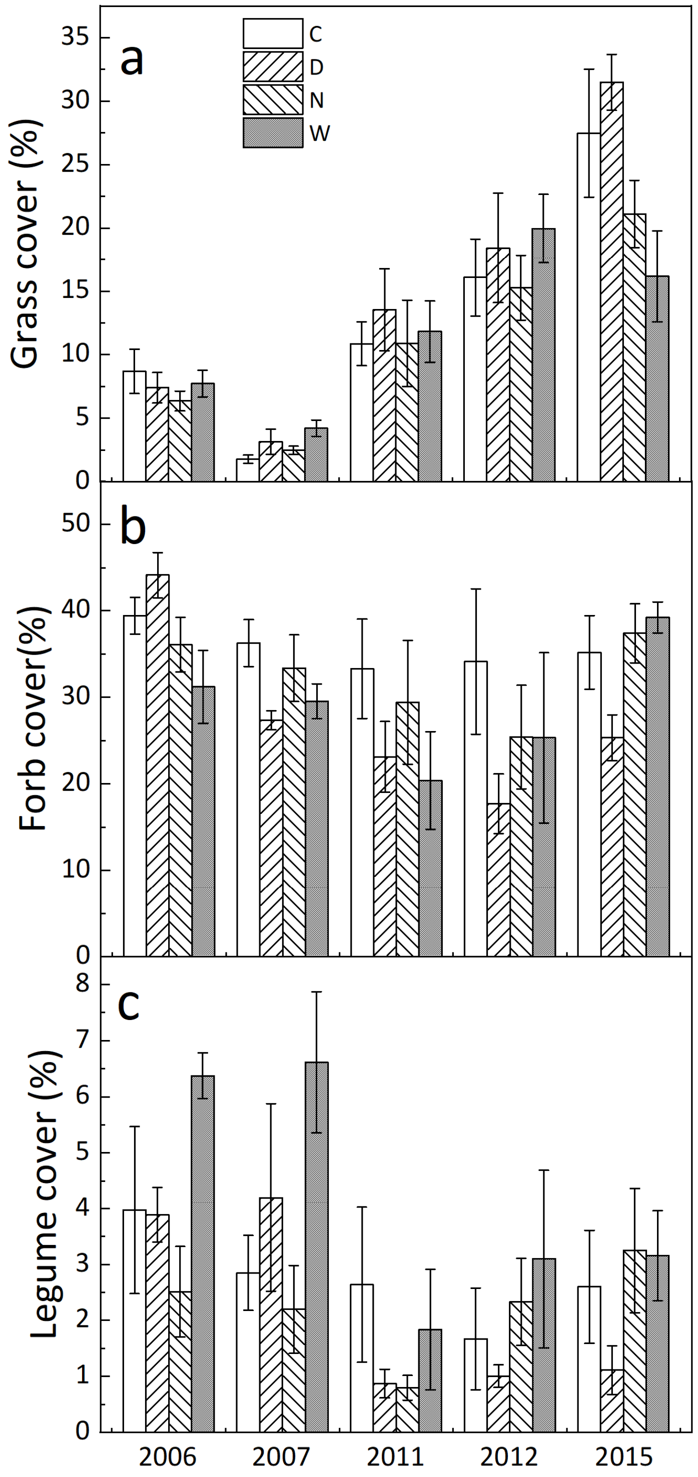

Figure S4 Dependences of the relative abundance of gram-positive (G+; a-d) and gram-negative (G-; e-h) bacteria on soil moisture (a, e), and plant cover of grasses (b, f), forbs (c, g) and legumes (d, h). Each data point represents the mean annual value in each plot. Solid line and shaded area describe linear regressions and 95% CIs.

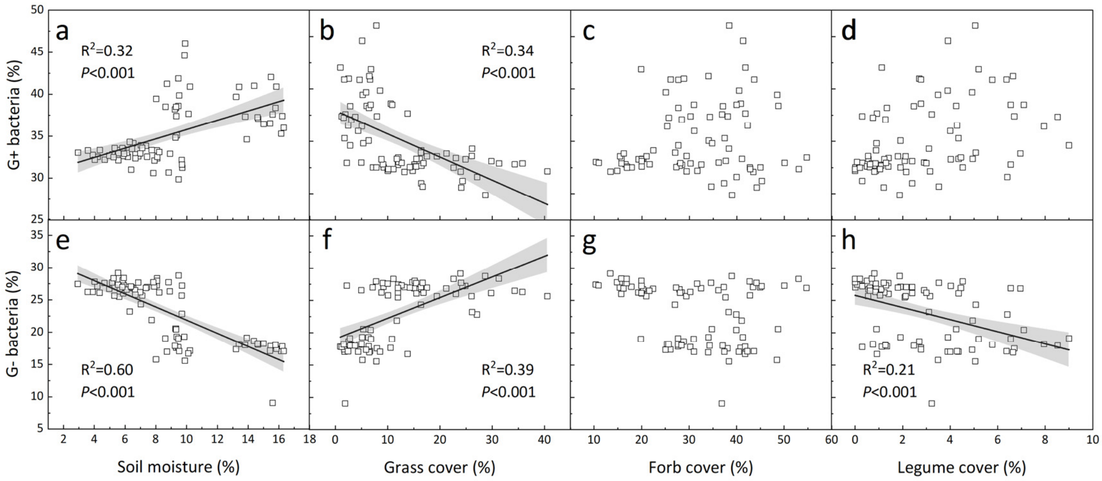

Figure S5. Dependences of the relative abundance of fungi on bacteria (a), G+ bacteria (b), G- bacteria (c), and G-/G+ ratio (d). Each data point represents the mean annual value in each plot. Solid line and shaded area describe linear regressions and 95% CIs.

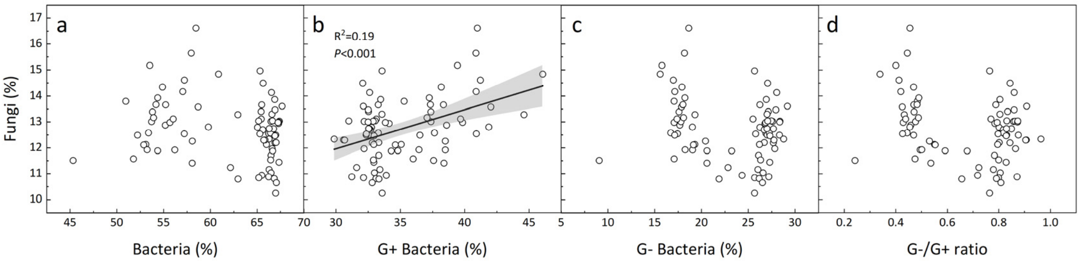

Figure S6 Dependences of the relative abundance of fungi (a, b) and the ratio of fungi to bacteria (c, d) on soil temperature (a, c) and moisture (b, d). Each data point represents the mean annual value in each plot in the short (2006 and 2007) and long term (2011, 2012 and 2015). Solid line and shaded area describe linear regressions and 95% CIs.

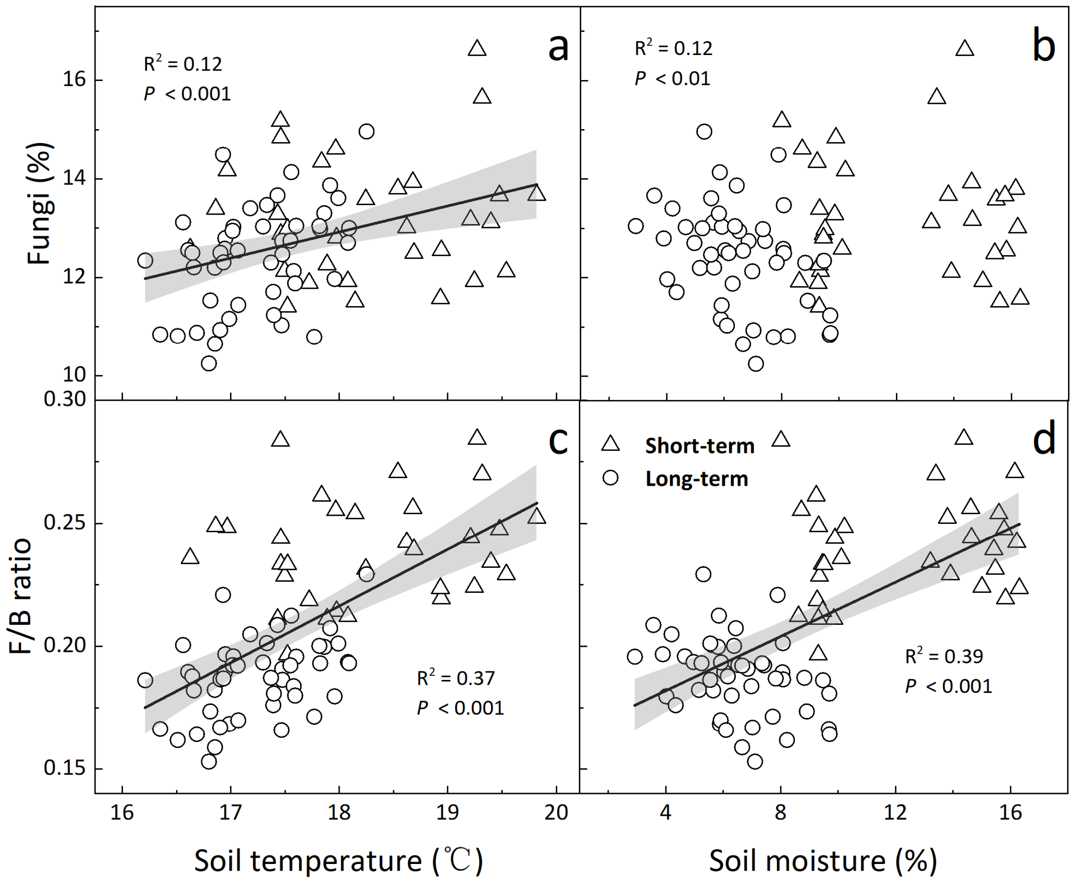

Figure S7 Dependences of grass (a, b), forb (c, d) and legume cover (e, f) on soil temperature (a, c, e) and moisture (b, d, f). Each data point represents the mean annual value in each plot in the short (2006 and 2007) and long term (2011, 2012 and 2015). Solid line and shaded area describe linear regressions and 95% CIs.

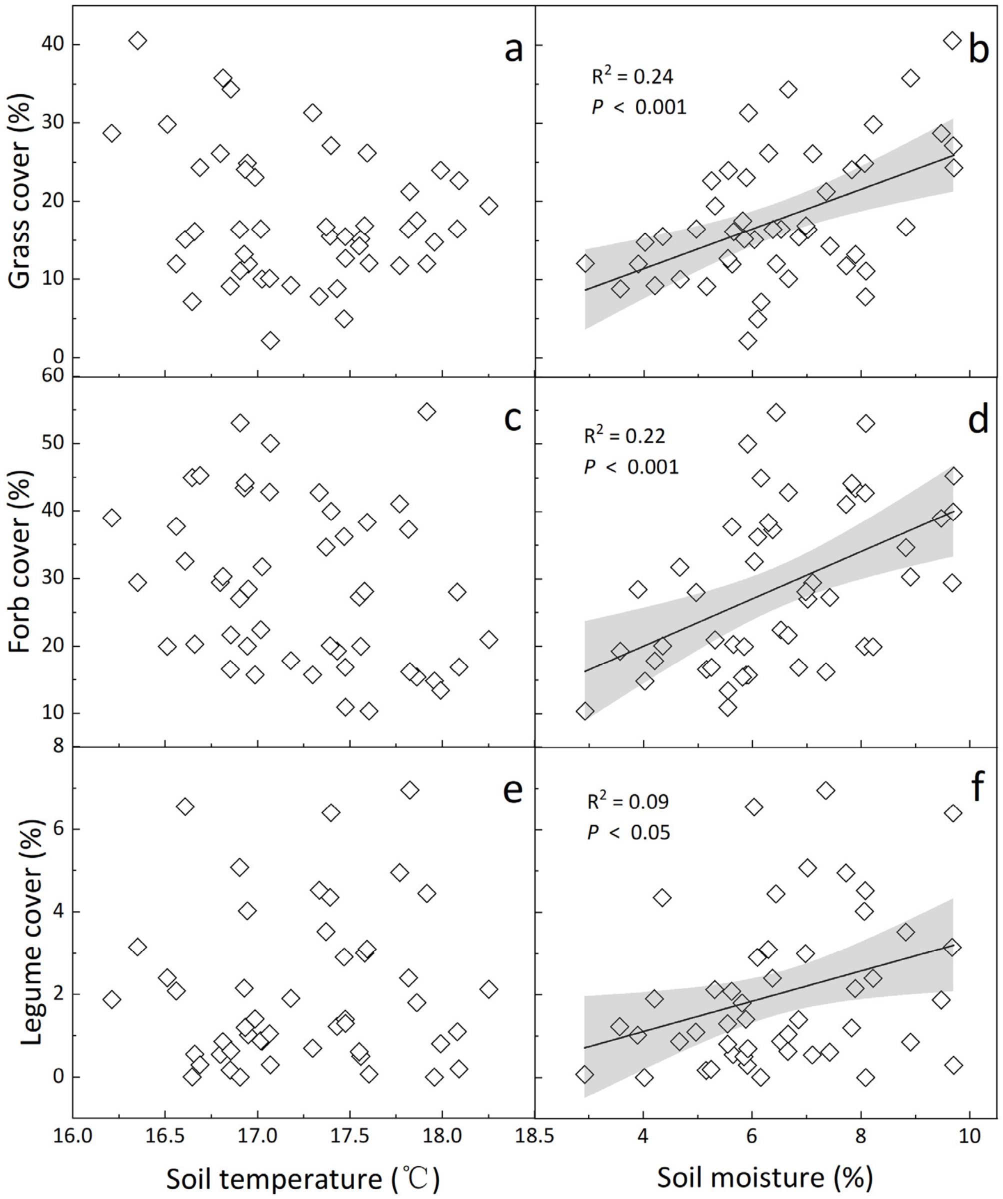

Figure S8 Dependences of warming-induced changes in the relative abundance of fungi (a-e) and the ratio of fungi to bacteria (f-j) on warming-induced changes in grass cover (a, f), forb cover (b, g), legume cover (c, h), soil temperature (d, i) and moisture (e, j). Each data point represents the mean annual value in each plot in the short (2006 and 2007) and long term (2011, 2012 and 2015). Solid line and shaded area describe linear regressions and 95% CIs.

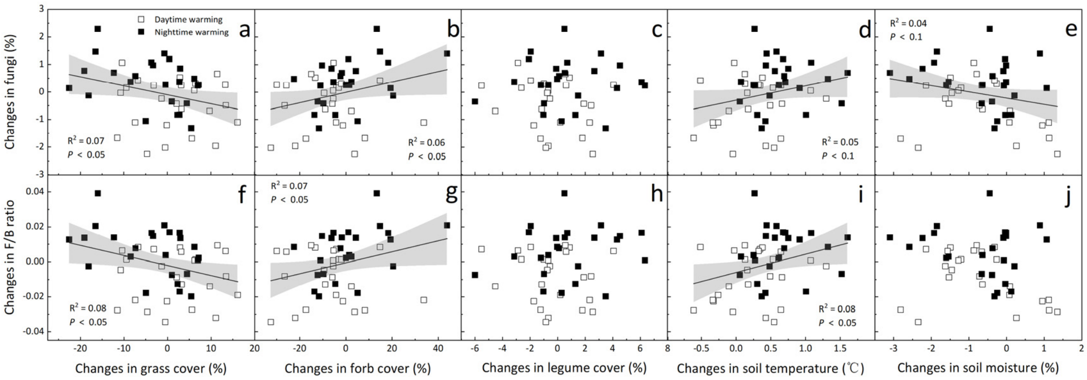

Supplement: Supplementary file 1 [file biology-12-00699-s001.zip › biology-2355199-supplementary.pdf]
